# Job Crafting in Nursing: A Conceptual Analysis for Theoretical Advancements in Nursing Practice

**DOI:** 10.1155/jonm/6599866

**Published:** 2025-08-17

**Authors:** Xi Yuan, Xuequn Yin, Xinmei Zhang, Zhengyu Ju

**Affiliations:** Department of Anesthesiology and Surgery, The First Affiliated Hospital of Soochow University, Soochow 215000, China

**Keywords:** concept analysis, crafting, job crafting, job crafting in nursing, nurses, nursing

## Abstract

**Aim:** To delineate a precise definition of job crafting within the nursing profession to deepen comprehension and stimulate progress in clinical practice and scholarly investigation.

**Background:** In the context of contemporary workplaces, job crafting is recognized as a multifaceted strategy for aligning employee roles with their capabilities and preferences. Nevertheless, its application in nursing, a field marked by high stress and complex demands, remains underexplored and lacks tailored assessment tools.

**Method:** We used Walker and Avant's systematic concept analysis method, which encompasses eight distinct steps. An exhaustive search for relevant English-language literature was performed across various databases, including PubMed, Web of Science, Wiley, CINAHL, and Scopus.

**Results:** Our review of 67 selected studies revealed the following defining attributes of job crafting in nursing: (1) proactive challenge engagement; (2) experience-informed and/or innovative behavior; (3) task, cognitive, and/or relational adjustments; and (4) demand–resource balance achievement. Antecedents were identified as intrinsic personal attributes and extrinsic organizational and societal influences. Conversely, while job crafting predominantly yielded positive outcomes, it could also sometimes result in adverse effects.

**Conclusion:** Through rigorous concept analysis, we established a well-defined understanding of job crafting in nursing, laying a solid conceptual foundation for future advancements in clinical applications and research endeavors.

**Implications for Nursing Management:** In light of this lucid concept, nursing managers can better comprehend the work behaviors and mechanisms of nurses. Accordingly, they can then develop tailored assessment tools to evaluate nurses' job crafting and devise appropriate strategies to facilitate it.

## 1. Introduction

In contemporary workplaces, employees frequently tailor their roles to align with their capabilities, needs, and preferences, a phenomenon recognized as job crafting [1]. Despite the absence of a unified definition, job crafting is generally operationalized through two dominant lenses: role-based and resource-based perspectives [2]. Role-based job crafting, initially posited by Wrzeniewski and Dutton [1], encompasses proactive employee actions to modify their work role boundaries and perceptions to suit their individual needs. This perspective categorizes job crafting into task, cognitive, and relational dimensions [1]. Conversely, resource-based job crafting, which stems from the job demands–resources model and was proposed by Tims et al. [3], views job crafting as employees' self-initiated efforts to balance job demands and resources. This framework delineates job crafting into three categories: augmenting job resources, elevating challenging job demands, and reducing hindering job demands [4].

Lichtenthaler and Fischbach [5] incorporated these definitions into a unified conceptual model, distinguishing between promotion-focused and prevention-focused job crafting on the basis of their respective beneficial and detrimental outcomes. Extending beyond individual dimensions, Lean et al. [6] introduced a group-level perspective on job crafting, emphasizing the expansion of task, relationship, and cognitive boundaries to the team level through collaborative efforts aimed at fostering a harmonious organizational climate and enhancing performance. Although extensive research has established a positive correlation between job crafting and employee performance, well-being, and organizational outcomes [5, 7, 8], job crafting remains a multifaceted concept, with its interpretation varying across industries and individual differences.

In the high-stakes field of nursing, where professionals are often confronted with high-stress scenarios and intricate patient-care demands, job crafting emerges as a critical strategy for bolstering work engagement and performance [9, 10]. Despite the burgeoning literature on job crafting, there is noticeable scarcity of research specific to the nursing context. Although initial studies have begun to explore the relevance and efficacy of job crafting behaviors within nursing, our understanding remains limited in several key areas. These include how nurses specifically engage in job crafting, the unique characteristics of job crafting in this profession, and the lack of tailored and validated instruments for measuring and assessing job crafting among nurses. A comprehensive understanding is thus imperative to harness the full potential of job crafting in nursing.

Concept analysis is a structured methodology for researchers to dissect and comprehend complex concepts thoroughly [11]. It has been recognized as a valuable tool for advancing both the theoretical and clinical development of the nursing discipline [12]. From this perspective, we posit that conducting a concept analysis of job crafting in nursing would provide a clear definition and framework for understanding job crafting within the context of the unique challenges inherent to nursing. Moreover, it addresses a significant research gap by offering a more nuanced understanding of how job crafting is applied by nurses. This insight can guide the development of measurement tools and strategies to promote its effective application. Consequently, the aim of this research is to delineate a precise definition of job crafting within the nursing context, thereby enhancing the understanding of job crafting in nursing. This will help catalyze progress in the development of measurement tools and the exploration of effective interventions related to job crafting in nursing, ultimately contributing to the advancement of nursing science.

## 2. Materials and Methods

### 2.1. Overview of the Concept Analysis

Concept analysis is a vital strategy for clarifying terms that are used in various fields but are often plagued by ambiguity. In the realm of nursing, there is frequent engagement with subjective behavioral concepts or those adapted from other disciplines that may not have clear-cut definitions. This ambiguity can result in cognitive distortions, impeding the progress of clinical practice and scholarly inquiry. Among the numerous techniques for concept analysis in nursing, the framework proposed by Walker and Avant [11] is notably prevalent.

According to Walker and Avant [11], concept analysis entails a thorough review of the semantics and application of a term or phrase, which lays the groundwork for theoretical construction. It promotes in-depth comprehension, logical demarcation, and accurate quantification of the concept under scrutiny. In our investigation, we embraced their methodology to dissect and delineate job crafting in nursing, with the goal of establishing a transparent conceptual structure for subsequent practical and investigative activities in this domain. The procedural steps of the Walker and Avant [11] approach encompass the following: (1) concept selection; (2) clarification of objectives for the analysis; (3) exploration of the concept's application; (4) ascertaining defining attributes and formulating operational definitions; (5) development of a prototypical example; (6) differentiation between marginal, analogous, and contrasting cases; (7) recognition of antecedents and consequences; and (8) establishment of empirical referents.

### 2.2. Data Collection and Selection Process

**Figure 1:**
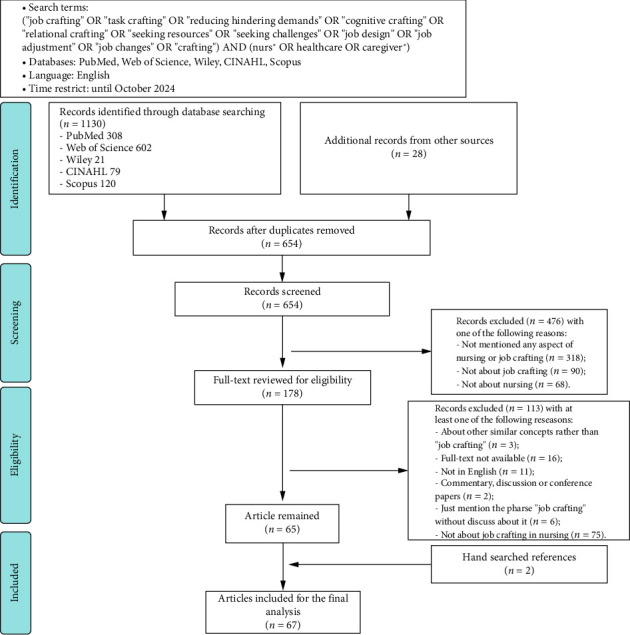
Search flowchart. Adapted from Moher, D., Liberati, A., Tetzlaff, J., Altman, D.G., and Prisma Group, 2009. Preferred Reporting Items for Systematic Reviews and Meta-Analyses: The PRISMA statement. PLoS Medicine, 6 (7), p.e1000097.

To ensure a thorough and exhaustive literature review, we used a comprehensive search strategy across multiple databases, including PubMed, Web of Science, Wiley, CINAHL, and Scopus. Our search terms were carefully selected on the basis of the existing definition of job crafting and insights from prior literature reviews. The keywords were “job crafting,” “task crafting,” “cognitive crafting,” “relational crafting,” “seeking resources,” “seeking challenges,” “reducing hinder demands,” “job design,” “job adjustment,” “job changes,” “crafting,” “nurs^∗^,” “healthcare,” and “caregiver^∗^,” and Boolean operators were used for precision. The search timeline was from the inception of each database to October 2024. Our inclusion criteria were restricted to full-text English-language publications, excluding conference abstracts, discussion papers, and commentaries. To complement our database search, we conducted a manual search of additional online resources and scrutinized reference lists to uncover any further relevant studies. The detailed search strategy is shown in Figure 1.

## 3. Results

The dataset comprised 1130 initial records. After the exclusion of 504 duplicates and the inclusion of 28 records from other sources, the relevance of the resulting 654 entries was screened using titles and abstracts. This led to 178 records undergoing full-text review. Upon rigorous full-text screening, 65 articles were deemed eligible for inclusion. We further examined the references of these articles to identify any additional pertinent literature. Finally, 67 articles were selected for final analysis, providing a robust foundation for our review.

### 3.1. Literature Review of the Concept

The term “job crafting” is not explicitly defined in standard dictionaries. In Merriam-Webster [13], “craft” as a verb is defined as “to make or produce with care, skill, or ingenuity.” Collins Dictionary [14] provides a similar definition, stating that something crafted is “made skillfully.” The term “job” is consistently defined across dictionaries as a piece of work, a specific task, or an individual's duty or function [15, 16]. In the context of nursing, the term refers to the profession or the duties of a nurse [17]. Drawing from these definitions, job crafting in nursing can be interpreted as the meticulous shaping of one's work tasks within the nursing profession.

Several definitions of job crafting in nursing have emerged from previous studies. Job crafting has been defined as the spontaneous behavioral changes initiated by nurses to align their personal goals with duties, thus enhancing job skills and motivation [18]. Yepes-Baldó et al. [19] defined nursing work crafting as an active behavior that both responds to and shapes the demands of the work environment. They emphasized that nursing work crafting is centered on the process of redesigning work experiences and altering work elements for nurses [19]. Empirical evidence suggests that job crafting positively influences nurses' work engagement, organizational commitment, and job satisfaction at the individual level [20]; furthermore, it reportedly improves mental health and care quality [19] and bolsters team cooperation and knowledge sharing at the team level, leading to enhanced team creativity [21].

### 3.2. Defining Attributes

In the realm of conceptual analysis, identifying defining attributes is a pivotal procedure. This process not only facilitates a lucid and accurate comprehension of the concepts at hand but also enables the discernment of one concept from another [11]. Attributes represent consistent characterizations of the inherent features of concepts as documented in the literature. Four attributes of job crafting in nursing have emerged from the literature: (1) proactive challenge engagement; (2) experience-informed and/or innovative behavior; (3) task, cognitive, and/or relational adjustments; and (4) demand–resource balance achievement.

#### 3.2.1. Proactive Challenge Engagement

The first defining attribute of job crafting in nursing is proactive challenge engagement. In the domain of nursing, job crafting emerges within specific contexts, particularly when confronted with formidable challenges. Research indicates that nurses are more inclined to engage in job crafting in work environments characterized by crisis situations (e.g., COVID-19) [22, 23], career shocks [24], emergencies [25–27], and conditions of high stress and workload [28, 29]. This conduct is not merely a natural response to environmental alterations; it is also a proactive stance taken by nurses in their quest for transformation. Amid these challenging scenarios, nurses capitalize on their strengths to react to environmental shifts and actively seek out or create opportunities to optimize the content and context of their work [25, 28]. This pattern of behavior, which is spontaneous, proactive, and reactive at the same time, equips nurses with the flexibility and adaptability necessary to navigate unpredictable and ever-changing work environments, thus enhancing the quality and efficiency of nursing care [22, 28].

#### 3.2.2. Experience-Informed and/or Innovative Behavior

The second key attribute of job crafting in nursing is the interplay between experience-informed and innovative behaviors. When nurses encounter challenges, they often engage in job crafting through the lens of their prior work experiences and performance [28]. Gordon et al. [30] reported that nurses can deepen their comprehension and application of job crafting by invoking memories of past learning experiences. Similarly, Wang et al. [27] observed that the diversity in research experience and knowledge across emergency department nurses influences their job crafting behaviors, particularly when dealing with research tasks. Furthermore, as described by Ghazzawi et al. [31], job crafting is also recognized as a creative adjustment behavior. In situations where past reflections fail to address current issues, nurses turn to their creative abilities, looking ahead and proactively adapting to change. ICU nurses, for example, can autonomously design and modify work processes, creating a supportive and transparent work environment that promotes subjective well-being [25]. Sahay and Dwyer [22] viewed job crafting during crises as improvisation, wherein nurses expand their roles and missions and forge new relationships to imbue their work with meaning. Ghazzawi et al. [31] found that nurses with higher creativity levels are more inclined to engage in job crafting, and Wu et al. [32] suggested that this engagement in creative job crafting further amplifies their creative capabilities.

#### 3.2.3. Task, Cognitive, and/or Relational Adjustments

The third attribute of job crafting in nursing pertains to the task, cognitive, and/or relational adjustments. This multifaceted process unfolds at both individual and collective levels, encompassing a comprehensive transformation across these three critical domains. In particular, task crafting includes the addition or subtraction of work elements, modification of work processes, training of new staff, participation in meetings, and undertaking of supplementary coordinative duties [28, 33, 34]. Cognitive crafting involves the reconstruction of nurses' perceptions regarding the significance of their work, the value of the nursing role, and organizational identification [22, 28, 34–36]. Relational crafting encompasses the establishment or modification of connections with patients, family members, colleagues, and societal resources [22, 28, 34, 37]. Furthermore, researchers have upheld the importance of cognitive crafting, with Wijngaards et al. [23] positing that cognitive changes are the initial occurrences in the job crafting process which dictate the direction of behavioral crafting in tasks and relationships, whereas Romeo et al. [38] viewed job crafting as a dynamic interplay between cognitive crafting and behavioral (tasks and relationships) crafting aimed at enhancing the quality of care.

#### 3.2.4. Demand–Resource Balance Achievement

Demand–resource balance achievement constitutes the fourth attribute of job crafting in nursing. Given the profession's high-demand, resource-constrained environments [39], nurses strategically deploy work adjustments (Attribute 3) to optimize resources and manage escalating demands [4]. Through these adaptations, nurses establish a sustainable balance state between job requirements and available resources—defining the core manifestation of this attribute. This achieved balance inherently reduces role tensions and fosters work engagement [40, 41]. As demonstrated by Golfenshtein et al. [29], nurse preceptors face distinctive demand–resource conflicts due to dual clinical–pedagogical responsibilities. Scarcity of temporal resources combined with excessive workloads elevates the risk of omitted nursing care. To address these challenges, they proactively implement structural resource reinforcements and demand impact moderation strategies. These interventions facilitate the attainment of demand–resource equilibrium, thus reducing care omissions and preserving high-quality patient outcomes [29]. Crucially, empirical analyses reveal nurses' consistent preference for resource-seeking approaches (e.g., requesting temporary staffing supplements) over demand-reduction tactics (e.g., lowering care standards) when establishing demand–resource balance, a state empirically associated with enhanced occupational well-being and care performance [30]. Thus, the attainment of demand–resource balance, representing the outcome dimension of job crafting, enables nurses to effectively navigate workplace challenges [31].

### 3.3. Antecedents

Antecedents refer to the underlying factors or events that are instrumental in the origin of a particular concept [11]. In the case of job crafting in nursing, the antecedents can be divided into two principal domains: inherent (intrinsic) and environmental (extrinsic) elements.

Inherent (intrinsic) elements pertain to the individual attributes of the caregiver, encompassing personal traits and dispositions. Empirical studies have shown that a positive mindset substantially influences job crafting. Notably, nurses with elevated self-efficacy and optimism perceive their work in a more favorable light, leading to enhanced job recognition [42]. This positive view not only leads to a greater appreciation for their jobs but also empowers them to make adjustments to their work on the basis of their personal strengths and needs [43–45]. Another study indicates that there is a positive correlation between the emotional stability of nurses and their level of job crafting [46]. Furthermore, life satisfaction, which is correlated with higher levels of happiness, is reportedly associated with increased job crafting among nurses [47]. Besides positive mindsets, Ghazzawi et al. [31] revealed that higher levels of creativity were associated with a greater inclination to engage in job crafting among nurses, and Bacaksiz et al. [48] identified a correlation between nurses' job crafting and their educational level.

Environmental (extrinsic) elements pertain to the broader organizational and societal frameworks within which the caregiver functions, highlighting the contextual influences that shape their role and actions. Research on environmental elements is extensive, with a primary focus on three distinct levels: the job, the organization, and society. At the job level, intrinsic job characteristics, such as position hierarchy, opportunities for promotion, and work-related stress, significantly influence nurses' propensity to engage in job crafting [44, 48, 49]. At the organizational level, several factors, such as the type of institution (ministry or private) and its operational model, and the degree of organizational empowerment granted to employees impact the job crafting behaviors of nurses [48, 50, 51]. Moreover, research indicates that leadership styles that are strength-based, shared, and supportive can create a conducive work environment and ensure the availability of high-quality job resources, thus facilitating job crafting among nurses [24, 32, 52, 53]. At the societal level, regional social resources, public health status, and the presence of emergencies (e.g., the COVID-19 epidemic) all influence the job crafting behavior of nurses [26, 27, 54, 55].

### 3.4. Consequences

Consequences refer to the outcome events that result from the execution of a concept [11]. Empirical evidence suggests that job crafting among nursing staff predominantly yields positive outcomes, notably by enhancing work performance and boosting occupational well-being [56–60]. Job crafting not only fosters greater work engagement among nurses [61–63] but also strengthens the bond between nurses and patients [23, 49, 64]. It encourages communication and sharing of knowledge among the nursing team, spurs innovation, streamlines workflow processes, and elevates both nursing performance and the quality of patient care [21, 56, 58, 65]. Concurrently, job crafting plays a significant role in bolstering nurses' well-being [66, 67], reinforcing a sense of organizational identification [68, 69], elevating job satisfaction and happiness [57, 59, 70], and mitigating burnout and the propensity for turnover [42, 71–75].

Nevertheless, certain researchers have observed some negative outcomes of nurses' job crafting. Srulovici et al. [76] pointed out that the job crafting strategy of increasing challenging demands cannot effectively improve the quality of nursing care for nurse mentors but will instead increase the instances of missed nursing care. Moreover, in times of crisis, job crafting behaviors could exacerbate the stress borne by nurses [26]. In addition, it has been noted that nurses contemplating leaving their positions may harbor persistent negative emotions throughout the cognitive crafting process [77].

### 3.5. Identify Model and Additional Cases

As a methodological tool, constructed cases differentiate critical attributes from peripheral features, thus clarifying the defining boundaries of conceptual constructs [11]. We presented a model supplemented by illustrative cases (borderline, related, and contrary) to demonstrate real-world manifestations of job crafting in nursing and to clarify the concept's scope and boundaries.

#### 3.5.1. Model Case

Model cases highlight all the attributes that are central to the concept, offering a clear illustration of how these defining attributes manifest collectively in a real-world or theoretical scenario [11].

James, a young male nurse in the operating theater, encountered a critical situation during a craniotomy when a patient experienced an intraoperative cardiac arrest. Swiftly, James coordinated with the surgical team to administer emergency resuscitation, including chest compressions. Following two defibrillation attempts, the patient regained sinus rhythm. In retrospect, James identified some challenges in the resuscitation process: The surgical bed's narrowness and the mattress's softness impeded effective chest compressions, potentially compromising patient safety and the success of resuscitation efforts. He recognized the pressing need to address this issue as a nurse to not only ensure patient safety but also safeguard the well-being of medical staff. Consequently, he embarked on a thorough review of the literature and sought the opinion of experienced seniors. Guided by these insights, James designed a mobile chest compression bedboard tailored for intraoperative resuscitation in the operating room. Following implementation, the bedboard significantly improved compression depth accuracy, dramatically shortened setup time, and substantially alleviated nurse-reported physical strain. These outcomes collectively achieved demand–resource balance by optimizing resuscitation effectiveness while conserving staff resources. The innovation received positive peer feedback and was adopted hospital-wide. Through this, James solidified his professional identity.

This model case meticulously demonstrates the four attributes of nursing job crafting as a causally sequenced process: James' proactive challenge engagement is evidenced through his immediate response to intraoperative cardiac arrest and subsequent identification of the resuscitation process limitations; his task, cognitive, and/or relational adjustments materialize in literature review and colleague consultations to reconceptualize the problem. These adjustments enable experience-informed and/or innovative behavior culminating in the customized bedboard design. Finally, demand–resource balance achievement manifests as optimized clinical efficacy (objectively improved compression accuracy), enhanced operational efficiency (significantly reduced setup time), and conserved human resources (substantially alleviated physical strain). This progression, wherein behavioral adaptations (Attributes 1–3) yield systemic equilibrium (Attribute 4), shows the operationalization of nursing job crafting. The case exemplifies all the defining attributes of job crafting in nursing, providing a compelling illustration of the concept's application in real-world work scenarios.

#### 3.5.2. Borderline Case

Borderline cases represent clinically ambiguous scenarios where the defining attributes manifest incompletely or asynchronously, challenging clear conceptual categorization [11].

Sarah, an experienced ICU nurse with 7 years of clinical practice, was promoted to preceptor in a teaching hospital grappling with 35% new graduate turnover. Proactively recognizing novices' struggles with ventilator management, she developed evidence-based quick-reference guides and initiated weekly 30-min coaching sessions. However, these uncompensated responsibilities were layered onto her existing 12-h shifts amid rising patient acuity. When workload intensified, Sarah made partial adjustments: delegating documentation tasks to novices (often requiring rework), cognitively reframing teaching as a “temporary sacrifice,” and avoiding peer collaboration to conserve energy. Although novice confidence scores improved by 15%, her personal medication errors increased by 40%, peer resentment escalated, and she developed chronic insomnia—culminating in medical leave for burnout before systemic solutions could be implemented.

This borderline case shows the critical limitations of partial job crafting: Sarah's proactive engagement (Attribute 1) and experience-informed innovations (Attribute 2) addressed novice training demands, and her superficial adjustments (Attribute 3) created illusory coping mechanisms. Crucially, her failure to achieve demand–resource balance (Attribute 4) manifested in quantifiable resource depletion—escalating clinical errors, eroded team cohesion, and personal health deterioration. The asymmetrical outcomes (15% novice confidence gain versus 40% personal error increase) reveal how isolated behavioral adaptations collapse without systemic equilibrium. Unlike successful crafting that synchronizes all four attributes, Sarah's compartmentalized efforts improved external demands while catastrophically neglecting internal resources, confirming job crafting as a noncompensatory process where balance achievement remains the keystone outcome determining sustainable efficacy.

#### 3.5.3. Related Case

Related cases depict phenomena that look similar to the target concept's attributes but actually serve fundamentally distinct purposes, facilitating differentiation of the target concept from adjacent constructs [11].

Emma is a nurse in a well-staffed intensive care unit. After the hospital introduced a new time-management system, she strictly complied. She adjusted her workflow by checking patients' vital signs and documenting care as scheduled. With this system, Emma found that her work demands better aligned with available resources, allowing her to efficiently fulfill her duties. However, Emma did not actively seek opportunities or challenges brought by the change. She did not use her personal experience or innovation to adapt; instead, she made adjustments purely for compliance and not to address patient-care challenges or from her own initiative.

This case shares superficial similarities with job crafting through task adjustments and improved demand–resource balance. Nevertheless, it fundamentally represents externally mandated procedural compliance. Unlike job crafting, which requires nurses' proactive engagement to align work with patient-care needs and personal resources, this case was driven by administrative directives. Emma's passive adjustments lacked self-initiated problem-solving, highlighting how top-down compliance differs substantively from authentic job crafting in achieving work equilibrium.

#### 3.5.4. Contrary Case

Contrary cases deliberately exclude all defining attributes, which help delineate the concept's boundaries and what it does not encompass [11].

Upon graduation, Taylor, a nursing postgraduate, joined a prestigious hospital with the initial goal of securing a position in administration with a focus on research endeavors. Contrary to her expectations, she was assigned to the emergency department for clinical nursing due to the hospital's staffing strategies. Like her peers, Taylor was responsible for the daily care of patients, including night shifts. Her advanced educational qualifications led to her being assigned additional research and mentorship tasks, which compounded her workload and induced significant stress, resulting in progressively deteriorating work performance. Despite the escalating challenges, Taylor did not initiate any interventions to address or alter her circumstances. Consequently, her initial passion for nursing waned, culminating in her decision to resign from her nursing role.

This case showed no attribute of job crafting in nursing. Taylor's lack of proactiveness to seek adjustments or changes when faced with a misalignment between her professional needs and actual job conditions resulted in the persistence of her workload–resource mismatch, adversely affecting her work performance and impacting her career development and personal well-being.

### 3.6. Operational Definition

Job crafting in nursing is defined as the dynamic process where nurses proactively respond to challenges, drawing on past experiences and/or integrating innovation, to adjust their tasks, cognition, and/or relationships. This process aims to achieve a balance between the demands of both patients and nursing staff and available resources, thus optimizing care delivery.

### 3.7. Empirical Referents

Empirical referents are a method of assessing the extent to which a concept exists or occurs, which provides researchers with a tool to measure and quantify the concept [11]. In the domain of nursing, job crafting research has predominantly employed questionnaires, with the job crafting scales authored by Tims et al. [4] and Slemp and Vella-Brodrick [78] emerging as particularly influential instruments.

Tims et al. [4] constructed a Job Crafting Scale grounded in the job demands–resources model, encompassing four dimensions with a total of 21 items. This scale has garnered international acclaim and has been translated into multiple languages, including Turkish [18], Spanish [10], and Chinese [79], reflecting its broad applicability and acceptance. Building upon the job crafting theory established by Wrzesniewski and Dutton [1], Slemp and Vella-Brodrick [78] formulated a Job Crafting Questionnaire (JCQ) encompassing 15 items across three dimensions: task, relationship, and cognitive crafting. Their JCQ's comprehensive scope has rendered it a staple in nursing job crafting research. In a subsequent development, Dvorak [80] offered novel perspectives on Wrzesniewski and Dutton's theory, resulting in the creation of an alternative scale comprising 21 items across these three dimensions, which, after translation into Chinese, has been extensively used in Chinese research contexts. In addition, Leana et al. [6] introduced the Individual and Collaborative Crafting Scale, pioneering the concept of “collaborative crafting.” However, this scale predominantly emphasizes task crafting and lacks explicit items related to relational and cognitive crafting.

Although the aforementioned scales have been widely adopted, they were not tailored for the nursing demographic. To address the unique requirements of the nursing profession and to enhance the assessment of team-based job crafting, Iida et al. [33] conducted qualitative interviews with nurses in two Japanese hospitals, leading to the development of the Team Job Crafting Scale for Nurses. This scale comprises 13 items across three dimensions: task crafting for team growth, cognitive crafting reflecting member respect and the significance of work, and relationship crafting for efficient information sharing. Although designed specifically for nurses, the scale's validity needs to be substantiated through extensive empirical research.

In summary, the empirical referents of job crafting in nursing have been extensively explored, with existing scales showing robust reliability and validity in studies. Researchers can select the most suitable measurement tools based on their study's design and observational objectives. However, these scales may not fully capture the nuances of job crafting specific to nursing, and there is a dearth of integrated measures that address both individual and team-level job crafting. Consequently, to foster advancements in nursing job crafting research, it is imperative to gain a comprehensive understanding of its unique characteristics and develop scales that are specifically calibrated for the nursing context.

## 4. Implications for Nursing Management

The introduction of job crafting into the nursing domain has sparked scholarly interest, with a plethora of research focused on its influencing factors and effects. Although job crafting has garnered recognition for its positive impacts on individuals and organizations, the absence of a clear definition within the nursing domain has impeded its research and development. A concept analysis of job crafting in nursing is vital for advancing nursing science and practice, offering a pathway to enhance the professional lives of nurses and ultimately contribute to the quality of patient care. However, the reliance on assessment tools from other disciplines has led to a shortfall of studies that capture the distinct nuances of job crafting within nursing. Consequently, these studies have not fully illuminated the unique aspects of job crafting in nursing, and the efficacy of promotion strategies has been compromised by their lack of specificity and impact, hindering the progression of research in this area.

**Figure 2:**
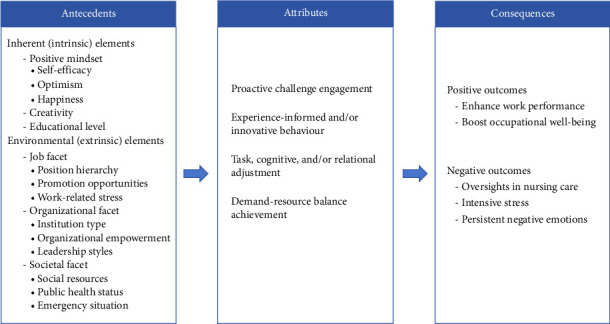
Conceptual model of job crafting in nursing.

Our exploration into job crafting in nursing (see Figure 2) represents a significant step toward a deeper understanding of how nurses can proactively mold their work to achieve higher levels of engagement and performance, thus enhancing their well-being and the overall efficacy of healthcare delivery. Future studies can build upon this foundation, refining relevant theories and devising more targeted and specialized assessment tools. This will empower researchers and nursing managers to better comprehend the work behaviors of nurses and the underlying mechanisms. Armed with this understanding, researchers and nursing managers can develop tailored assessment tools to measure nurses' job crafting behaviors accurately. Furthermore, effective interventions that address the influencing factors, which may include optimizing resource allocation and improving the nursing work environment, can be designed to inspire nurses to engage in job crafting. Such initiatives not only foster the professional development and well-being of nurses but also elevate the overall quality of nursing services.

## 5. Conclusion

A thorough review of scholarly works describes job crafting in nursing as a strategy in which nurses actively engage in the challenge and adjust existing work tasks, cognitions, and/or relationships based on experience and/or innovation to align resources and demands. Job crafting in nursing can be influenced by individual attributes, organizational structures, and broader societal influences, leading mostly to positive results such as improved job performance and heightened occupational well-being, though occasionally causing more stress and negative feelings. Various job crafting scales are at the disposal of researchers to gauge the extent to which nurses engage in job crafting. In this manuscript, we used Walker and Avant's methodological framework for concept analysis to delineate a robust conceptual foundation for job crafting within the nursing profession. This foundation is intended to underpin both clinical practice and pave the way for future scholarly exploration in this niche area of study.

## Data Availability

The authors confirm that the data supporting the findings of this study are available within the article.
